# Deciphering the Iron Metabolism and Ferroptosis in Diabetic Wound Healing

**DOI:** 10.2174/0115733998352547250107065356

**Published:** 2025-02-28

**Authors:** Yashi Feng, Yunchang Cao, Yun Ou-Yang, Wuxiang Wang, Shaolong Feng

**Affiliations:** 1 The Guangxi Key Laboratory of Environmental Exposomics and Entire Lifecycle Health, School of Public Health, Guilin Medical University, Guilin, 541100, China;; 2 Department of Molecular Biology, School of Intelligent Medicine and Biotechnology, Guilin Medical University, Guilin, 541100, China;; 3 The Library, University of South China, Hengyang, 421009, China;; 4 The State Key Laboratory of Organic Geochemistry, Guangzhou Institute of Geochemistry, Chinese Academy of Sciences, Guangzhou, 510640, China

**Keywords:** Ferroptosis, iron metabolism, diabetic wounds, wound healing, diabetic hyperglycemia, cellular migration

## Abstract

Diabetic-related complications, such as delayed and incomplete wound healing, are an increasing concern in the realm of public health. Ferroptosis represents an innovative variant of cellular demise. Ferroptosis is currently thought to be an essential factor in the process of diabetic wound recovery. This article, therefore, examines the novel function and mechanism of ferroptosis in the repair of diabetic wounds. Diabetic hyperglycemia can induce a healing process that disrupts the function and activity of cells, thereby impeding the repair of diabetic wounds. Ferroptosis may be accelerated in diabetic lesions due to protracted low-level inflammation and oxidative stress induced by elevated glucose, according to the available evidence. As a result, the buildup of ferroptosis impedes cellular migration and proliferation, amplifies oxidative stress and the inflammatory response, and ultimately interferes with the wound-healing process. By regulating the expression of factors linked to iron mortality, this substance expedites wound healing and fosters angiogenesis in diabetic rodents. Moreover, new perspectives on the difficulties and outlooks related to ferroptosis in the context of diabetic wound healing are provided, thereby contributing to the progression of understanding in this field.

## 1 INTRODUCTION

Diabetes mellitus (DM) poses a significant global public health challenge, affecting 500 million individuals worldwide. According to data from the International Diabetes Federation in 2019, this figure is projected to increase to 755 million by 2045 [1]. Diabetic ulcers are a highly consequential complication of DM, impacting 19-34% of individuals with the disease and imposing substantial financial strain on the community [2, 3]. Non-healing diabetic wounds are the predominant factor contributing to the increased rate of amputations in people with DM, leading to elevated mortality rates and placing a substantial socio-economic burden [4]. In severe instances, non-healing wounds resulting from diabetes may ultimately necessitate amputation. According to a study, the annual financial burden resulting from medical expenses and reduced productivity associated with diabetic wounds is anticipated to surpass $200 billion [5]. Importantly, amputation is associated with a substantial mortality rate of 16.7% after 12 months and exceeding 50% after 5 years, surpassing the mortality rates observed in various cancer instances. The study revealed that in addition to renal tubular cells (HK-2 cells), cultured human dermal fibroblasts (HDF) and vascular endothelial cells exhibited diminished cellular function and aberrant expression of ferroptosis-related proteins, Excessive reactive oxygen species, along with lipid peroxidation byproducts, led to a significant down-regulation in the expression of the Ferritin Heavy Chain 1 (FTH1) protein within these cells [6, 7]. Wound recovery was accelerated in a diabetic rat model by directly administering ferroelastic-1, a ferroptosis inhibitor, or desferrioxamine, an iron chelator [8]. According to these reports, ferroptosis might contribute to the formation of diabetic wounds and presents a promising target for diabetic wound treatment.

Several investigations have indicated a substantial association between a decrease in iron levels and the onset and progression of type 2 diabetes mellitus (T2DM) and its related repercussions [9]. Ferroptosis is a distinctive form of cellular demise that sets it apart from apoptosis, necrosis, and autophagy due to its distinct morphological, biochemical, and genetic characteristics [10]. The mechanisms governing iron-induced cell death involve complex interactions among various cellular pathways, including the oxidation of polyunsaturated fatty acids (PUFAs) and phospholipids at optimal levels, disturbance of iron homeostasis, and compromise of antioxidant systems [11, 12]. Ferroptosis has been observed to be linked with a range of human illnesses. Ferroptosis plays a crucial role in the development of diverse multi-system disorders, encompassing, but not restricted to, respiratory diseases, disorders of the nervous system, cardiac conditions, breast-related ailments, gastric disorders, hepatic ailments, pancreatic conditions, renal disorders, intestinal illnesses, reproductive system disorders, dermatological conditions, and musculoskeletal disorders.

In spite of recent progress in diabetic wound healing research, there remains a deficiency in comprehending the fundamental pathological and molecular mechanisms involved. Additionally, the effectiveness of current treatment techniques is not deemed sufficient [13]. Currently, a growing body of research indicates that the occurrence of iron deficiency anemia markedly impacts the diabetic wound-healing process. This work provides a comprehensive analysis of the intricate regulatory processes behind diabetic wounds. It offers compelling evidence, both direct and indirect, indicating the engagement of ferroptosis in the advancement and resolution of diabetic wounds. Consequently, this study provides a significant contribution to the identification of innovative therapeutic targets and medications.

## 2 TRADITIONAL WOUND HEALING MECHANISMS AND FERROPTOSIS RESPONSE MECHANISMS

### 2.1 Traditional Wound Healing Mechanisms

The conventional wound healing process consists of four distinct phases: hemostasis, inflammation, proliferation, and remodeling, functioning in a meticulously regulated manner where the phases collaborate to restore injured tissue. During the initial phase of wound formation, the hemostatic phase establishes a blood clot *via* platelet aggregation and coagulation to avert hemorrhage and create a preliminary barrier. This is followed by the inflammatory phase, during which leukocytes migrate to the wound site to clear infection sources and necrotic tissue, establishing a sterile environment for subsequent repair. In the proliferative phase, fibroblasts, epithelial cells, and endothelial cells proliferate and migrate within the wound region, forming new tissue and supporting it through angiogenesis. Finally, in the remodeling phase, granulation tissue is gradually replaced by mature fibrous tissue, with collagen fibers reorganized to enhance tissue strength, resulting in a stable scar. In healthy individuals, this wound-healing process generally proceeds efficiently. However, in patients with chronic conditions like diabetes mellitus, healing is often delayed or incomplete due to factors such as uncontrolled inflammation, poor blood supply, and oxidative stress, leading to chronic, hard-to-heal wounds [14].

### 2.2 Iron Metabolism

Ferroptosis is a unique iron-dependent cell death process that differs fundamentally from apoptosis, necrosis, autophagy, and pyroptosis in its molecular pathways, triggers, and cellular effects. Unlike apoptosis, which is a programmed cell death involving caspase activation and mitochondrial cytochrome c release, ferroptosis is initiated by lipid peroxidation due to iron accumulation and oxidative stress, independent of caspase activity or DNA fragmentation. In contrast to necrosis, ferroptosis does not involve cell membrane rupture or immediate inflammatory responses, making it a more regulated process that specifically targets lipid metabolism and iron homeostasis [7]. Autophagy, another type of cell death, serves as a catabolic process where cells recycle their components under stress. While autophagy can interact with ferroptosis pathways, especially in terms of iron metabolism regulation, it generally functions to protect cells rather than induce cell death. In some cases, autophagy may contribute to ferroptosis by degrading ferritin, which increases free iron and promotes lipid peroxidation, thereby linking autophagy with ferroptosis in certain pathological contexts [15]. Pyroptosis, characterized by gasdermin-mediated pore formation in the cell membrane, is an inflammatory form of cell death distinct from ferroptosis. Triggered by pathogens or danger signals, pyroptosis activates inflammasomes and promotes inflammation through cytokine release, which is notably absent in ferroptosis. This inflammatory response is beneficial in infections but can exacerbate chronic conditions, making ferroptosis, which lacks a similar pro-inflammatory profile, a more suitable target for managing chronic diseases like diabetes [16]. In conclusion, ferroptosis represents a distinctive mechanism of cell death that diverges significantly from apoptosis, autophagy, necrosis, and pyroptosis, both in terms of its molecular triggers and its impact on cellular health. Its regulation of lipid peroxidation and iron metabolism without immediate inflammatory consequences positions ferroptosis as a promising therapeutic target for chronic oxidative stress-related conditions, including diabetic wounds and other complications of diabetes [17].

Iron, an essential nutrient for living organisms, performs a crucial function in various biological processes, including the transport of oxygen, generation of energy, immune response, replication of deoxyribonucleic acid (DNA), and the tricarboxylic acid cycle (TCA) [18, 19]. Moreover, iron is essential for the survival of cells. Iron exists in two different oxidation states, namely ferrous iron (Fe^2+^) and iron oxide (Fe^3+^) [20]. Dietary iron primarily resides in the form of Fe^3+^, and its absorption takes place within the intestinal epithelial cells [21]. Within the process of circulation, the primary binding of dietary iron occurs with transferrin through the formation of Fe^3+^ complexes. These complexes are subsequently conveyed through the cell membranes by means of transferrin receptors [22]. Couplers are internalized by cells through the process of endocytosis [23]. In the context of acidic endosomes, the process involves the liberation of Fe^3+^ from the coupling and subsequent reduction to Fe^2+^. This Fe^2+^ is subsequently transported from the endosomes to the cytoplasm with the participation of Six Transmembrane Epithelial Antigen 3 (STEAP3) of the prostate, aided by the divalent metal transporter protein 1 (DMT1) [24]. Iron is later internalized within endosomes through the activity of transferrin receptor 1 (TfR1) and is then conveyed to the cytoplasmic lysosome *via* DMT1. In the cytoplasm, iron can exist either in a free state or bound to serum ferritin, forming unstable iron pools (LIPs). These labile iron pools (LIPs) can be enclosed by autophagic lysosomes, a process facilitated by nuclear receptor coactivation factor 4 (NCOA4). Following degradation, substantial quantities of Fe^2+^ are liberated [25]. This Fe^2+^ functions as a vital iron source and plays a pivotal role in the regulation of iron metabolism (Fig. **1**) [26-31].

### 2.3 Lipid Metabolism

Lipid peroxidation, a chemical process instigated by free radicals, serves not only as a fundamental mechanism but also as a distinctive feature of ferroptosis. Lipid peroxidation entails the biochemical process by which lipids containing carbon-carbon double bonds, specifically unsaturated fatty acids within cellular membranes, are specifically attacked by oxidants like free radicals [32-35]. Products resulting from lipid peroxidation include primary lipid hydroperoxides (LOOH) and subsequent reactive aldehydes such as malondialdehyde (MDA) and 4-hydroxynonenal (4HNE). These compounds exhibit an elevation in concentration during the process of ferroptosis [20]. Polyunsaturated fatty acids (PUFAs) are the primary subjects of lipid peroxidation [36]. Among them, polyunsaturated fatty acids like arachidonic acid (AA) and adrenergic acid (ADA) are the most vulnerable to lipid peroxidation during ferroptosis [37, 38]. Arachidonic acid (AA) and adrenergic acid (ADA) are the primary polyunsaturated fatty acids (PUFAs) that trigger ferroptosis. Dixon observed that the acyl-coenzyme A synthase long-chain family, particularly those responsible for the synthesis of ACSL4 and LPCAT3, play crucial roles in the ferroptosis pathway through the introduction of multiple mutations in haploid cells [39]. Moreover, lipoxygenase (LOX) is involved in the mediation of ferroptosis [39]. Multiple studies have demonstrated that suppressing or inhibiting the mentioned three synthases can impede the initiation and progression of ferroptosis [40, 41].

Iron accumulation and lipid peroxidation are key metabolic characteristics driving ferroptosis. Genes associated with iron and lipid metabolism play crucial roles in modulating ferroptosis through both direct and indirect mechanisms, influencing iron accumulation and lipid peroxidation. Glutathione (GSH), the primary non-enzymatic antioxidant, acts as a cofactor for glutathione peroxidase 4 (GPX4), an enzyme that converts harmful lipid peroxides into less reactive alcohols, thus maintaining cellular redox balance [42, 43]. Disruption in this balance, marked by increased lipid peroxide levels and iron accumulation, initiates ferroptosis, which damages cellular structures such as lipoproteins, proteins, and DNA [44]. PUFAs, including omega-3 and omega-6 fatty acids, are especially prone to lipid peroxidation due to the presence of multiple double bonds, which render them highly reactive. For example, linoleic acid (LA) and arachidonic acid (AA) are particularly susceptible to peroxidation, leading to cellular damage and ferroptosis. Studies indicate that lipid radicals formed during peroxidation react with reactive oxygen species (ROS) to create lipid peroxyl radicals, which further propagate the lipid peroxidation chain reaction [45]. Interestingly, monounsaturated fatty acids (MUFAs), such as oleic acid and palmitoleic acid, exhibit protective effects by competing with PUFAs for incorporation into phospholipids, reducing membrane susceptibility to peroxidation. This competition lowers the overall rate of lipid peroxidation and mitigates ferroptosis [37, 46]. Moreover, enzymes like DECR1 play a significant role in PUFA β-oxidation. Inhibition of DECR1 expression has been shown to elevate PUFA levels in mitochondria, intensifying oxidative stress and lipid peroxidation, which underscores the importance of lipid metabolism regulation in ferroptosis [47, 48]. Finally, the lipid peroxidation process involves both enzymatic and non-enzymatic pathways. Non-enzymatic peroxidation is typically mediated by free radicals generated through Fe^2+^ reactions with ROS, whereas enzymatic peroxidation primarily involves lipoxygenases (LOXs) and cytochrome P-450 oxidoreductase (POR). Recent studies, including lipidomic analyses, show that POR can enhance ferroptosis by promoting the peroxidation of membrane-bound PUFAs across multiple cell types. This dual pathway underscores the complexity of lipid peroxidation in ferroptosis and highlights potential therapeutic targets for modulating ferroptosis in pathological conditions [49, 50].

The interplay between iron metabolism and lipid peroxidation is a feedback loop that amplifies ferroptotic cell death. Excess free iron drives ROS production, which, in turn, initiates and propagates lipid peroxidation. Lipid peroxidation itself consumes PUFAs, creating lipid radicals that react further with oxygen to propagate the peroxidation chain reaction, leading to extensive damage across cellular membranes. The oxidative burden imposed by iron and lipid peroxidation in ferroptosis is exacerbated by diabetes-induced metabolic stress, which often results in antioxidant depletion. When GPX4 and other antioxidant defenses are overwhelmed, cells are unable to prevent the accumulation of lipid peroxides, leading to membrane disruption and ferroptotic cell death. This process is particularly detrimental in diabetic wounds, where persistent oxidative stress and impaired healing are already challenges [51].

### 2.4 Antioxidant Mechanisms

#### 2.4.1 GPx4 Pathway

GPx4 is classified as the fourth constituent within the family of glutathione peroxidase (GPxs) enzymes, characterized by their inclusion of selenocysteine and dependence on glutathione [52]. The GPX4 gene plays a crucial role in the advancement and maintenance of various physiological processes and is widely acknowledged as a prominent inhibitory gene of ferroptosis [53]. The enzyme GPX4 facilitates the lipid peroxidation process, playing a critical role in averting the accumulation of lipid peroxides and the subsequent onset of ferroptosis [54]. The GPX4 pathway exerts regulatory control over Ferroptosis through multiple mechanisms; 1) The enzymatic function of GPX4 enables the transformation of lipid peroxides into their respective alcohols, thereby mitigating their toxicity and reducing the likelihood of inducing ferroptosis. Inhibition of the enzymatic activity of GPX4 leads to the accumulation of lipid peroxides, thereby triggering the onset of ferroptosis. 2) Preservation of cellular membrane integrity: The cellular membranes are highly vulnerable to lipid peroxidation, a process that can result in membrane impairment and subsequent initiation of ferroptosis. The GPX4 protein is pivotal in maintaining the structural integrity of cell membranes by reducing the levels of lipid peroxides within these membranes. 3) The control of iron metabolism is essential in the context of ferroptosis, as iron plays a central role in initiating lipid peroxidation *via* the Fenton reaction [54]. The control of iron metabolism is managed by GPX4, which operates by binding to iron and impeding its participation in the Fenton reaction. In general, the GPX4 pathway plays a crucial role in governing ferroptosis by actively reducing lipid peroxidation, preserving membrane integrity, and modulating iron metabolism.

#### 2.4.2 Ferroptosis Suppressor Protein 1 (FSP1) Pathway

Research findings indicated that the responsiveness of various cell lines to GPX4 inhibitors exhibits significant variability, implying the existence of unknown mechanisms that regulate the down-regulation of ferroptosis, which extend beyond the scope of GPX4 [55]. Through the application of genes *via* genome-wide CRISPR-Cas9 screens, scholars have successfully discerned FSP1 as an additional pivotal element in the development of resistance to ferroptosis [56, 57]. Originally recognized as apoptosis-inducing factor (AIF)-like mitochondrion-associated inducer of death (AMID) or Apoptosis-inducing factor mitochondria-associated 2 (AIFM2, also identified as FSP1), FSP1 was the initial gene assigned to ferroptosis [58]. In comparison to AIF, FSP1 is predominantly found in the cytoplasmic lysate and demonstrates a potential affinity for binding to the outer mitochondrial membrane. However, it does not possess the extended N-terminal mitochondrial targeting sequence observed in AIF [59]. FSP1, a flavoprotein oxidoreductase, is postulated to exert its pro-apoptotic role *via* impeding redox activity. FSP1 has a binding affinity towards DNA, the nicotinamide cofactor, and a modified form of flavin known as 6-hydroxy-FAD. When it forms a complex with double-stranded DNA (*e.g.*, bacterial and (retroviral) viruses). The antagonistic nature of its action as an oxidoreductase would result in the buildup of ROS [58]. FSP1 was initially recognized as a gene that reacts to p53, a transcription factor involved in programmed cell death, and is commonly referred to as recombinant p53 responsive gene 3 (PRG3). Further investigations have substantiated the notion that the expression of FSP1 exhibits resistance towards ferroptosis while not displaying the same resistance towards apoptosis [60]. Follow-up studies have supplied further proof, suggesting that myristoylation augments the gathering of FSP1 at the cell membrane. Once localized at the plasma membrane, FSP1 functions as a redox enzyme and a lipophilic free radical trapping antioxidant. Its enzymatic activity involves the reduction of catalyzes the regeneration of ubiquinone (Coenzyme Q10) to panthenol, thereby effectively preventing the peroxidation of PUFA within the lipid bilayer. This mechanism ultimately leads to the inhibition of ferroptosis [57].

#### 2.4.3 The relationship between the GPx4 Pathway and the FSP1 Pathway

GPx4 and FSP1 are two critical pathways that modulate ferroptosis, exhibiting both complementary functions and distinct mechanisms in the prevention of this form of cell death. GPx4 successfully mitigates ferroptosis by diminishing phospholipid peroxides *via* GSH depletion and obstructing the progression of the lipid peroxidation cascade. GPx4 activity is essential for cellular antioxidant defense and significantly inhibits ferroptosis in several cell types. The inhibitory action of GPx4 relies on a sufficient supply of glutathione; hence, cells become more vulnerable to ferroptosis when GSH is depleted or GPx4 is inactivated. Unlike GPx4, FSP1 prevents ferroptosis by a mechanism that does not rely on glutathione [61]. FSP1 employs NAD(P)H as an electron donor to convert coenzyme Q10 (CoQ10) into coenzyme Q10H2 (ubiquinol), therefore diminishing lipid peroxidation. The ferroptosis inhibition of FSP1 is independent of GSH, offering supplementary protection during glutathione deficiency. This complementarity enables FSP1 and GPx4 to collaborate in inhibiting ferroptosis, thereby offering multiple layers of cellular protection [56].

Recent findings indicate that the connection between GPx4 and FSP1 demonstrates complexity in specific cancer cells. Research indicates that certain cancer cells depend on FSP1, rather than GPx4, to prevent ferroptosis under conditions of elevated oxidative stress. This indicates potential variations in the reliance of distinct cell types on GPx4 and FSP1. Moreover, it has been demonstrated that FSP1 overexpression might result in heightened antioxidant defenses in specific cell types and may disrupt the normal apoptotic process, thereby enhancing cellular resistance to pharmaceuticals. Consequently, the functions of GPx4 and FSP1 in the regulation of ferroptosis may be influenced by cellular type and environmental factors, and these contradictory results present a hurdle for the future implementation of ferroptosis-targeted therapeutics [62].

## 3 MECHANISMS OF FERROPTOSIS IN DIABETIC WOUNDS

### 3.1 Diabetic Wound Mechanism

Wound healing is an intricate and ever-changing physiological process that serves a critical function in safeguarding the body against a multitude of detrimental external influences. It encompasses a series of sequential and interrelated processes, including hemostasis, inflammation, proliferation, and lysis/remodeling [63]. The wound-healing process is initiated by a sequence of intricately coordinated events that coordinate the progression of the aforementioned distinct yet overlapping phases of wound healing [64]. After hemostasis, the injury enters an inflammatory phase where various cells, including neutrophils, macrophages, keratinocytes (KCs), endothelial cells (ECs), endothelial progenitor cells (EPCs), and fibroblasts, organize and collaborate [65, 66]. Concerning the synthesis and control of various cytokines and growth factors, these cells are actively involved. Monocytes, which subsequently transform into macrophages, are credited with the production of pro-inflammatory cytokines such as Interleukin-1β (IL-1β), Tumor necrosis factor-α (TNF-α), Interleukin-6 (IL-6), and Vascular endothelial growth factor (VEGF). These cytokines are found in both diabetic and non-diabetic settings. Transforming growth factor-β (TGF-β), VEGF, and insulin-like growth factor-1 (IGF-1) are included. Aside from T and B cells, neutrophils are notable producers of IL-1β and TNF-α, along with keratinocytes, fibroblasts, mast cells, and endothelial cells. Additionally, they play a role in the generation of vascular endothelial growth factors, specifically IGF-1 and TGF-β [67]. Macrophages are vital to the process of healing. The epigenetic code is modified by hyperglycemia and oxidative stress, resulting in alterations to the regulation and polarization of macrophages. The imbalance in macrophage polarization significantly contributes to the delay in wound healing [68, 69]. Diabetes incurs significant expenses, with approximately one-third of the costs associated with the management of diabetic foot disease, particularly chronic, non-healing diabetic foot ulcers. Consequently, much effort has been devoted to elucidating the etiology of diabetic wounds and exploring novel therapeutic strategies. A novel area of focus is macrophage polarization and its significance in diabetic wound healing. Diabetic wounds demonstrate aberrant and sustained M1 (pro-inflammatory) macrophage polarization, while normal wounds show a transition to M2 (pro-healing) macrophages approximately three days post-injury. We examine the determinants that affect macrophage polarization, with an emphasis on those that promote M2 macrophage polarization, alongside prospective therapeutic strategies that partially address macrophage polarization in diabetic wound environments. A significant portion of the research has focused on mitigating hyperglycemia and facilitating the neutralization or diminished expression of pro-inflammatory cytokines, which are crucial in the generation of M1 macrophages. The care of diabetic wounds may necessitate a multimodal strategy encompassing the regulation of underlying diabetes and hyperglycemia, topical interventions, and the prevention of secondary infections and inflammation. A dynamic field of current research is the function of macrophages in the healing of diabetic wounds. Macrophages originate from monocytes, which are classified into three subpopulations: classical macrophages (CD14++CD16-), intermediate macrophages (CD14++CD16+), and nonclassical macrophages (CD14++CD16++) [68]. The milieu to which monocytes are exposed will dictate the polarization of macrophages into the aforementioned subgroups. This is significant as each type exerts a distinct influence on its environment. Classical macrophages, or M1 macrophages, are deemed proinflammatory due to their activation by proinflammatory cytokines, including tumor necrosis factor (TNF) and lipopolysaccharide (LPS), and their subsequent production of proinflammatory cytokines such as interleukin (IL)-12 and IL-23, along with reactive oxygen species (ROS) [68]. Conversely, nonclassical macrophages, referred to as M2 macrophages, are regarded as pro-healing or resolving macrophages. They are activated by anti-inflammatory cytokines, including IL-4 and IL-10, and secrete growth factors such as transforming growth factor (TGF)-β and insulin-like growth factor (IGF) [68]. A comprehensive review has been performed on the timing and function of macrophages in normal wound healing, as well as the disruptions or modifications in diabetic wound healing [70-73]. In typical, non-diabetic wound healing, M1 macrophages are predominant from day 1 to day 3, followed by a switch to M2 macrophages, which peak around day 7 of the healing process [74]. The primary macrophage phenotype in diabetes wounds is often M1, regardless of the time elapsed since the initial injury, and is a crucial element contributing to the chronicity of diabetic wounds [75]. The conversion of M2 macrophages to M1 has been linked to compromised wound healing, inadequate angiogenesis, and diminished collagen deposition [76]. It is important to acknowledge that while the aforementioned procedure delineates a definitive account of macrophage predominance in wound healing, it is a dynamic process influenced by the surrounding microenvironment [77]. Considering that M2 macrophages are predominantly regarded as pro-healing, one hypothesis posits that wound healing would be expedited if only M2 macrophages were present, hence inhibiting the polarization of all M1 macrophages. Moreover, the macrophage type present in the wound is crucial, as is the timing of their presence; M1 macrophages facilitate the phagocytosis of germs and tissue debris while releasing inflammatory cytokines that enhance the healing process [68]. The impairment of wound healing in diabetes is multifaceted. Chronic inflammation is believed to be a factor contributing to the development of this injury. Recent studies have emphasized the significance of macrophage polarization in this process. The failure of macrophages to transition from the M1 phenotype to the M2 phenotype approximately three days post-injury is a significant difference between normal and diabetic wound healing. In diabetic patients, various factors have been discovered that perpetuate the activation of M1 macrophages, primarily hyperglycemia, which induces a chronic inflammatory state that favors the M1 macrophage phenotype. Numerous novel medicines are being formulated and evaluated to inhibit specific inflammatory signals, thereby preventing M1 macrophage activation. Both systemic and local therapy may be required to adjust the inflammatory profile, including macrophage polarization, to manage the chronic wounds associated with diabetes, a common and severe condition.

The delay in wound healing in diabetic patients is attributed to complex molecular mechanisms. Diabetic wounds arise when continuous inflammation or atypical variables hinder wound healing. They progress into chronic wounds that demonstrate challenges in healing due to several circumstances. Primarily, hypoxia is the principal factor impeding the healing of diabetic wounds. Oxygen is a crucial regulator of wound healing processes, including skin cell proliferation, granulation, re-epithelialization, angiogenesis, and tissue regeneration [78]. Following injury, hypoxia escalates in the affected region due to heightened oxygen consumption resulting from disrupted blood flow and inflammatory response, alongside diminished oxygen supply [79]. This results in a notable reduction in oxygen use, leading to hypoxia linked to elevated blood glucose levels [80]. Clinical studies indicate that the hyperoxic conditions linked to hyperglycemia and tissue hypoxia might permeate non-healing diabetic wounds, leading to protracted wound healing [81]. Hypoxia extends the duration of successful wound healing by amplifying the inflammatory response and elevating oxygen-free radical levels. A continuous oxygenation system was developed, comprising oxygen-releasing microspheres and a hydrogel designed to scavenge ROS. It was demonstrated to expedite the healing process by continuous oxygenation and reactive oxygen species scavenging. It specifically augments the proliferative phase and diminishes pro-inflammatory cytokine expression [82].

Secondly, diabetic wounds are marked by a diminished capacity for local angiogenesis. The process relies on a careful equilibrium between encouraging the growth and proliferation of blood vessels during standard wound healing and facilitating their maturation and quiescence; however, the pathophysiology of diabetes mellitus significantly disturbs this balance, hindering normal wound healing and tissue regeneration. Research indicates that VEGF-A protein and PDGF are markedly diminished in the wounds of db/db mice relative to normal controls. This implies that diabetes impairs angiogenesis during wound healing, hence complicating proliferation [83-85]. Consequently, facilitating diabetic wound healing through the augmentation of angiogenesis at the wound site has emerged as a prominent strategy.

Third, in contrast to typical wound healing, diabetic wounds have a persistent inflammatory phase marked by an abundance of macrophages [86]. In diabetic wounds, the polarization of M1 macrophages is dysregulated and prolonged, whereas in normal wounds, macrophages transition to M2 macrophages approximately three days post-injury [87]. Moreover, heightened myelopoiesis in hematopoietic stem cells, attributable to the diabetic microenvironment, has been documented to lead to increased circulating levels of Ly6CHi monocytes. These monocytes infiltrate the wound and differentiate into macrophages exhibiting M1- like characteristics, such as interleukin-1β (IL-1β) and tumor necrosis factor-α (TNF-α), both of which impede keratinocyte migration and hinder wound healing [88].

Fourth, irregularities in extracellular matrix buildup and remodeling primarily influence the proliferative and remodeling phases of diabetic wound healing. The extracellular matrix largely functions as a supporting element in the wound-healing process. Nonetheless, the architecture and functionality of the extracellular matrix in diabetic wounds are markedly compromised due to fibroblast dysfunction and augmented interstitial spaces between collagen fibers [89]. Moreover, elevated glucose levels result in ECM proliferation, thickness, and glycosylation [90]. It also results in the formation of advanced glycosylation end products (AGEs), which are commonly identified in extracellular matrix proteins in diabetes individuals [91]. The mechanical support offered by ECM proto-fibers to capillary sprouts during angiogenesis in diabetic wounds is diminished. These impacts cause structural and functional anomalies in the extracellular matrix, potentially resulting in delayed wound healing.

In conclusion, diabetic wounds encounter more significant healing difficulties than other types of wounds. They are distinct from ordinary wounds because of the presence of hypoxia, poor angiogenesis, persistent inflammation, and aberrant extracellular matrix, which contribute to a protracted healing process.

Studies have indicated that diabetic animal models exhibit compromised synthesis of factors crucial for healing, such as growth factors, macrophages, and neutrophils, as well as impaired migration and proliferation of keratinocytes and fibroblasts [92, 93]. Furthermore, there have been accounts of compromised angiogenic reactions and complications in microvasculature. Since the identification of ferroptosis, numerous recent studies have concentrated on the prevention and treatment of tumors [10]. In recent years, the role of ferroptosis in diabetic wounds has attracted great interest as our understanding of ferroptosis has increased [94]. Depletion of GPX4 and GSH, increased iron levels, and heightened lipid peroxidation are shared characteristics in both ferroptosis and diabetes [95]. Numerous studies have linked several diabetic complications to elevated glucose levels, which induce alterations in upstream coregulatory elements, resulting in diminished iron overload and antioxidant capacity through various pathways [96, 97]. The present research indicates that autophagy controls ferroptosis by adjusting cellular iron balance and the generation of reactive oxygen species [98]. Ferroptosis and iron phagocytosis are evident in diabetic complications arising from inherent disruptions in cellular metabolic pathways, such as defective iron metabolism, directly linked to the initiation and advancement of diabetic complications [99].

### 3.2 Iron Metabolism and Diabetic Wounds

Elevated iron levels and heightened lipid peroxidation are prevalent characteristics of the ferroptosis response and diabetes mellitus, with a correlational relationship between ferroptosis and the onset of diabetic complications. Diabetic wounds represent a significant complication, impacting 19%-34% of diabetic patients. Diabetic refractory wounds are typically distinguished by the presence of neuropathy, delayed re-epithelialization, localized ischemia and hypoxia, and persistent wound inflammation [86]. Researchers have continued to investigate the mechanisms that contribute to the development of diabetic ulcers and search for the most effective treatments to address this issue. The gradual discovery of the impact of ferroptosis on diabetic wound healing has occurred in recent years. It is believed that the presence of high glucose(HG)accelerates the progression of diabetic wounds, worsens the occurrence of ferroptosis, and increases the delay in wound healing [7, 100].

Studies showed that the persistent inflammation and oxidative stress in diabetic wounds can exacerbate delayed healing through iron-dependent lipid peroxidation. Cells in high-glucose environments exhibit elevated levels of ROS and lipid peroxidation products, triggering ferroptosis. Ferroptosis inhibitors, such as Ferrostatin-1, can improve diabetic wound healing by reducing the expression of inflammatory markers [7]. Moreover, chronic hyperglycemia leads to an accumulation of lipid peroxidation products and disrupts iron metabolism, further delaying wound healing and contributing to the underlying mechanism of diabetic ulcers [17]. Platelet-rich plasma (PRP) has been shown to significantly promote the healing of diabetic ulcers by inhibiting ferroptosis, reducing inflammatory factors, enhancing antioxidant defenses, and improving angiogenesis [51]. Additionally, autophagy-mediated reduction of Fe^2+^ production and regulation of iron metabolism help to enhance cellular repair functions in diabetic wounds, thereby promoting the healing process [15]. Therefore, ferroptosis plays a crucial role in delayed diabetic wound healing, and strategies targeting its inhibition hold promise as new approaches and interventions for treating chronic diabetic wounds. These findings collectively suggest that the suppression of ferroptosis positively influences wound healing by reducing inflammatory responses, enhancing fibroblast migration and proliferation, and promoting angiogenesis, thereby improving overall tissue regeneration. The potential therapeutic application of ferroptosis inhibitors for treating diabetic wounds is promising, as they may offer an effective approach to managing chronic, non-healing wounds by addressing multiple physiological aspects of wound repair simultaneously. Orientin is a C-glycosylated flavonoid extractable from foods like Dr. Tea and passion fruit. It possesses anti-inflammatory, anticancer, and antioxidant properties [101]. Research indicates that the Nrf2/GPX4 signaling pathway is significantly linked to diabetes and the reparative processes of damage resulting from mitochondrial dysfunction and ferroptosis. The precise mechanism by which Orientin operates in diabetic wounds remains incompletely elucidated. We expect that Orientin, *via* activating the Nrf2/GPX4 pathway in diabetic wound healing, may inhibit HG-induced ferroptosis and ameliorate mitochondrial dysfunction. The use of Orientin will achieve this. According to the findings, Ori seems to facilitate the nuclear translocation of Nrf2, the expression of GPX4, and proteins related to angiogenesis, as well as the adhesion and migration of HUVEC. *In vivo* studies have demonstrated that Ori therapy enhances DM wound healing, angiogenesis, and the up-regulation of GPX4 expression. RSV is a naturally occurring phytochemical exhibiting diverse biological and pharmacological characteristics [102]. Research indicates that it exerts a protective impact against diabetic microvascular problems, with numerous research presenting persuasive findings about the advantages of utilizing RSV to mitigate diabetes complications. This study indicated that RSV can mitigate damage in both *in vivo* and *in vitro* diabetic models. In the diabetic rat model, the expression of the angiogenesis-promoting factor VEGF and the vascular endothelial cell marker CD31 was elevated, diabetic wound healing was expedited, and the expression of ferroptosis-related marker proteins was modified. RSV is proposed to enhance diabetic wound angiogenesis and healing by suppressing ferroptosis. Nrf2 is pivotal in mitigating oxidative and electrophilic stress, as well as regulating cellular redox status and energy metabolism [103, 104]. Multiple investigations have demonstrated that many proteins and enzymes that inhibit lipid peroxidation and, consequently ferroptosis are target genes of Nrf2 [105, 106]. Recent findings indicate that RSV mitigates oxidative stress through the modulation of the Nrf2 pathway [107]. Initially, transcriptome analysis of treated endothelium cells demonstrated that the Nrf2 mRNA level in the AGEs+RSV group was markedly elevated compared to the AGEs group, a finding that Western Blotting subsequently corroborated. Consequently, we deduce that RSV suppresses ferroptosis in vascular endothelial cells. It may facilitate angiogenesis and enhance wound healing in diabetic injuries. The activation of Nrf2, a gene implicated in oxidative stress, has been suggested as a potential strategy for avoiding ferroptosis [108, 109]. Nrf2 is a crucial regulator of iron-induced cell death and has been demonstrated to modulate many genes, including the GPX4 gene [110]. Research by Li, Liang, and colleagues revealed that the activation of the Nrf2/GPX4 signaling pathway can significantly diminish ferroptosis [111, 112]. The significant findings were reported in the journal Cell [61]. The Nrf2/GPX4 signaling pathway is crucial for sustaining active mitochondrial function and stability. Orientin, a C-glycosylated flavonoid, is found in fenugreek, which exhibits diverse biological activities and therapeutic advantages, such as anti-inflammatory, antidiabetic, antioxidant, and autophagy-inducing effects [113, 114]. Molecular biology research indicates that Orientin is intricately associated with oxidative stress, mitochondrial function, and the Nrf2 signaling pathway [115, 116]. Conversely, there is a paucity of research exploring the impact of Orientin on diabetic wound healing and the underlying mechanisms involved. This study aimed to evaluate the effects of Orientin on HUVEC subjected to high glucose, as well as the mechanisms underlying these effects and the beneficial influence of Orientin on a diabetic mouse wound model. The data indicate that Orientin effectively inhibits ferroptosis by diminishing oxidative stress, improving mitochondrial function, and regulating the Nrf2 signaling pathway. These data suggest that Orientin may serve as a potential therapeutic alternative for diabetic wounds. *In vitro* investigations have demonstrated that elevated glucose concentrations lead to reduced survival and migration of HUVECs, alongside heightened levels of ROS, lipid peroxidation products, and proteins linked to ferroptosis in cellular fractions. Nonetheless, therapy with Ori or Fer-1 mitigated the adverse consequences of elevated glucose levels. Moreover, in the streptozotocin-induced diabetes mouse model, wound healing was markedly accelerated in diabetic mice administered Ori or Fer-1. This was followed by reduced levels of reactive oxygen species and ferroptosis, alongside heightened activation of the antioxidant signaling pathway, including Nrf2/GPX4. The results indicate that Ori mitigates ROS, therefore preventing ferroptosis, and enhances the synthesis of pro-angiogenic proteins, thus facilitating improved healing of diabetic wounds.

Non-healing diabetic wounds are characterized by senescence, chronic inflammation, elevated apoptosis, and reduced cell proliferation. The TP53 gene is a crucial tumor suppressor that regulates cell proliferation, senescence, DNA repair, and apoptosis. While p53 is crucial for cell cycle regulation, its function in initial tissue repair seems to be harmful. In standard cutaneous injuries, p53 levels temporarily decrease and then swiftly revert to baseline levels. However, in diabetic wounds, prolonged activation of p53 impedes wound healing through two distinct pathways: 1) activation of the p53-p21-retinoblastoma (RB) axis, which arrests the cell cycle, and 2) upregulation of the cGAS-STING and nuclear factor-kappaB (NF-κB) cascades, which induces a ferroptosis response. Furthermore, p53 interacts with other metabolic pathways, including glycolysis, gluconeogenesis, oxidative phosphorylation, and autophagy. In diabetic wounds, p53 may induce metabolic reprogramming, potentially altering macrophage polarization. Persistent p53-mediated dermal inflammation in diabetic ulcers. In diabetic wounds, keratinocytes, fibroblasts, and macrophages display a pronounced inflammatory condition [117]. In these wounds, various variables, including TNF-α, p53, FOXO1, S100A2, MIF, Caspase 3/7, IL-6, PICP, and CCF are raised and may correlate with p53. Moreover, anomalous activation of specific inflammatory pathways can be detected in diabetic wounds, wherein p53 may be implicated. Activation of p53 suppresses the AKT-mediated autophagy route and the Wnt-β-catenin pathway while inducing DNA damage to activate the cGAS-STING pathway. The suppression of p53 by 53BP1 mitigates the inflammatory damage resulting from DNA damage. Wounds associated with diabetes. TNF-α (Tumor Necrosis Factor Alpha), FOXO1 (Forkhead Transcription Factor O1), S100A2 (previously CaN19 or S100L), MIF (Macrophage Migration Inhibitory Factor), IL-6 (Interleukin 6), PICP (Collagen C Peptide), CCF, AKT (Protein Kinase B), mTOR (Mammalian Target of Rapamycin), cGAS-STING (Cyclic GMP-AMP Synthase-Stimulator of Interferon Genes) GMP-AMP-interferon gene stimulator, SASP senescence-associated secretory phenotype. Chronic skin senescence in diabetic wounds depends on p53. Usp7, along with HG-induced PARP-1, Caveolin-1, and PTRF, activates p53, which triggers cellular senescence and apoptosis. The up-regulation of p53 in diabetic wounds may significantly contribute to cellular senescence in diabetic skin lesions. Senescent cells in diabetic wounds secrete substantial quantities of SASP, which triggers the activation of inflammatory responses and produces cell cycle arrest. Pioglitazone, curcumin, and senolytics suppress p53 and mitigate the progression of diabetic wounds. USP7 ubiquitin-specific protease 7, HG hyperglycemia, PARP-1 ADP-ribose polymerase-1, PTRF polymerase I and transcription release factors, pioglitazone (Piog), and calcium silicate (CS). p53-mediated pan-apoptosis in diabetic wounds. The hyperglycemic milieu in diabetic wounds triggers the activation of p53, which then activates the xCT-GSH pathway, resulting in enhanced iron-induced apoptosis in vascular endothelial cells [117]. Conversely, the overexpression of FABP4 in macrophages facilitates the activation of SIRT1, resulting in the ubiquitination of p53 and, eventually, the activation of NLRP3-mediated thermal apoptosis. These characteristics indicate the existence of pan-apoptosis in diabetic wounds, resulting in delayed healing of such wounds. p53 in several cellular strata of diabetic wounds. p53 exhibits distinct functions across several cortical areas in diabetic wounds. The MAPK and P53 pathways are involved in apoptosis, proliferation, differentiation, and inflammation of fibroblasts in diabetic wounds. The presence or lack of TNF-independent p53 in diabetic wounds significantly influences the progression of apoptosis and inflammation in such wounds. The lack of lncH19 or increased levels of TNF-α and Cav-1-PTRF-1 activates p53, thereby influencing GDF15 and bcl-2, resulting in diminished fibroblast proliferation and impacting macrophage growth. In vascular endothelial cells of diabetic wounds, USP7 promotes the ubiquitination activation of p53, resulting in cellular senescence. Moreover, platelet-activating protein in diabetic wounds promotes the induction of senescent components, including p53, through CD47. In adipocytes within diabetic wounds, p53 enhances energy expenditure in brown adipocytes, consequently suppressing their proliferation. Furthermore, p53 promotes insulin resistance in white adipocytes; however, temporary inhibition of p53 expression triggers autophagy, which mitigates insulin resistance in these cells [117]. The research carried out by Song *et al.*, presented proof that fibroblasts in diabetic wounds among the elderly display resilience to cell death induced by iron [118]. Furthermore, this resistance may be effectively treated by increasing the expression of NCOA4, leading to accelerated wound healing. Research has demonstrated that fibroblasts that have become senescent due to several factors, such as radiation, replication, and carcinogenesis, have a significant level of resistance to ferroptosis. Furthermore, in aging fibroblasts stimulated by an HG environment, there is a substantial buildup of ferritin and a considerable decrease in NCOA4 expression. In such circumstances, ferritin attaches to iron within cells, so diminishing the amount of free iron that is accessible. Consequently, cells develop resistance to ferroptosis as a consequence of the decrease in available iron [119]. The inhibition of ferroptosis triggers the activation of the PI3K/AKT signaling pathway in diabetic wounds, leading to the control of oxidative stress and inflammation. Consequently, this mechanism contributes to the improvement of diabetic wound healing [119]. We performed an experiment involving streptozotocin-induced diabetic rats and observed that administering Fer-1 to diabetic rats led to accelerated wound healing. This was accompanied by a decrease in the infiltration of inflammatory cells and the secretion of VEGF, along with an elevation in the activation of the anti-inflammatory PI3K-AKT signaling pathway. Thus, the administration of medications to inhibit ferroptosis diminishes inflammation and boosts the production of pro-angiogenic substances, ultimately leading to improved wound healing [7]. VEGF is a prominent growth factor that promotes the development of new blood vessels by augmenting the proliferation, migration, and viability of cells lining the inner walls of blood vessels [120]. The interaction between fibroblasts and endothelial cells is vital for the phenomenon of angiogenesis [121, 122]. The inadequate healing of diabetic wounds can be ascribed to reduced proliferation of vascular endothelial cells and migration of epithelial cells, coupled with the existence of intense inflammatory conditions in the epidermis [123]. Proliferating cell nuclear antigen (PCNA) is a protein involved in DNA replication. It exhibits heightened activity during the late G1 and S phases of the cell cycle, rendering it a dependable indicator of cellular nuclear proliferation [124]. Our findings indicate that the use of Fer-1 led to an increase in VEGF and PCNA expression, a reduction in the infiltration of inflammatory cells and levels of pro-inflammatory cytokines, and an improvement in wound healing in the injured tissue of diabetic rats. Ultimately, through the utilization of *in vitro* and *in vivo* diabetes models, we have demonstrated that exposure to HG conditions results in excessive oxidative stress production in fibroblasts and vascular endothelial cells. Consequently, this leads to lipid peroxidation and ferroptosis. The administration of Fer-1 suppressed ferroptosis, resulting in the activation of the PI3K-AKT signaling pathway. This, in turn, led to a reduction in inflammation and an enhancement in cell migration and proliferation, ultimately fostering wound healing in diabetic rats [7]. Macrophages play a pivotal role in the wound healing process. In the later stages of inflammation, M1 macrophages undergo a progressive transformation into M2 macrophages, actively facilitating wound healing. In their *in vitro* investigation, Zhou *et al.* found that an excess of iron significantly influenced the expression of M1 macrophage markers, including IL-1β, TGF-α, and cluster of differentiation 86 (CD86). However, markers such as arginase-1 (Arg-1) and cluster of differentiation 206 (CD206) did not exhibit significant differential expression in M2 macrophages. This suggests that an excess of iron promotes the polarization of M1 macrophages. Iron overload induces the acetylation of p53 by increasing the production of ROS, subsequently leading to the polarization of M1 macrophages [125]. Additionally, iron-laden cells possess the capability to induce the release of high mobility group box chromosomal protein 1 (HMGB1), a protein that promotes the differentiation of pro-inflammatory macrophages [126, 127]. Significantly, a study on spinal cord injuries revealed that an excessive accumulation of iron caused a transformation of M2 macrophages into M1 macrophages [128].

In an artificial laboratory model, increased glucose levels resulted in heightened levels of ROS, products of lipid peroxidation, and proteins related to cell death caused by iron. Additionally, it led to decreased cell survival and migration. Moreover, the administration of Fer-1 mitigated the aforementioned consequences induced by elevated glucose levels [7]. Furthermore, a prior investigation discovered a notable decrease in the expression of the FTH1 protein in renal tubular cells (specifically HK-2 cells) when exposed to an HG environment [6]. Recent research has indicated that skin repair cells, namely HDF and vascular endothelial cells when cultivated in an HG environment, demonstrate impaired cellular activity along with abnormal production of excessive ROS, lipid peroxidation products, and proteins associated with ferroptosis. Within the HG environment, the levels and activity of the intracellular GPX4 protein are notably reduced, resulting in an excessive buildup of ROS and Fe^2+^. These substances are believed to be crucial factors that initiate ferroptosis (Fig. **2**) [129, 130].

### 3.3 Lipid Peroxidation and Diabetic Wounds

Inhibiting the expression of ACSL4 and LPCAT3 reduces the accumulation of substrates for lipid peroxidation within cells, thereby hindering the progression of ferroptosis [40]. Dramatically, the expression of ACSL4 was notably elevated, whereas the viability of cells in the lower thylakoid of HG was dramatically diminished [96]. Within this biological pathway, the upregulation of ACSL4 expression in response to HG levels ultimately triggers an increase in ROS, which in turn initiates the process of lipid peroxidation. Ultimately, this sequence of events leads to the onset of ferroptosis. Iron overload causes the polarization of M1 macrophages. Iron overload triggers p53 acetylation through the promotion of ROS release, leading to the polarization of M1 macrophages [125]. Diabetic wounds often have a predominance of M1 macrophages in the wound area, while the presence of M2 macrophages is minimal or absent. As a result, this causes persistent inflammation and impaired wound healing [87]. Crucially, ACSL4, and not other ACSLs, modifies the responsiveness of cells to iron-induced cell death by influencing the composition of lipids [40]. Reducing ACSL4 increases resistance to ferroptosis [131]. The increased expression of FTH1 escalated the occurrence of lipid peroxidation in endothelial cells and facilitated the death of these cells [132]. The presence of HG resulted in an elevation in the formation of both total and mitochondrial ROS in endothelial and fibroblast cells. Lipid peroxidation indicators, including 4-hydroxynonenal (4-HNE) and malondialdehyde (MDA), are prevalent in diabetic wounds. Lipid peroxidation indicators, including 4-HNE and MDA, are prevalent in diabetic wounds. Lipid peroxidation products comprise early lipid hydroperoxides (LOOH) and subsequent reactive aldehydes, such as MDA and 4HNE, which elevate during ferroptosis [133]. During the initiation step, ROS/RNS radicals interact with PUFA, extracting allyl hydrogens and generating lipid radicals. During the propagation phase, lipid radicals interact with oxygen to generate lipid peroxyl radicals, which subsequently react with PUFA to produce new radicals and lipid hydroperoxides. In the termination phase, antioxidants or lipid radicals can inhibit propagation by donating hydrogen atoms to lipid peroxyl radicals, leading to the generation of non-radical products. Lipid hydroperoxides, owing to their instability, may breakdown into secondary compounds. 4-HNE and MDA are the principal products among many reactive aldehydes [134]. 4-HNE, the most prevalent and toxic aldehyde resulting from lipid peroxidation, has been linked to the onset of various diseases, including cardiovascular diseases, neurological disorders, inflammatory bowel disease, diabetes mellitus, rheumatoid arthritis, and multiple cancer types [135-142]. Moreover, 4-HNE interacts with macromolecules (proteins, lipids, and nucleic acids) and modulates several signaling pathways, including Nrf2, NF-κB, PKC, and ERK [143]. Furthermore, 4-HNE is involved in various biological processes, including oxidative stress, inflammation, cell proliferation, and cell death [144-147]. MDA is frequently utilized as a biomarker for evaluating lipid peroxidation [148]. Moreover, MDA can interact with amino acids to form several adducts, including MDA-lysine and MDA-glycine [149, 150]. The MDA-acetaldehyde protein adduct, malondialdehyde-acetaldehyde (MAA), has been implicated in several disorders, including pancreatitis, atherosclerosis, rheumatoid arthritis, and inflammatory bowel disease. Moreover, it resulted in an augmentation of the production of MDA, a byproduct of membrane lipid peroxidation. However, the effects of HG on MDA production were greatly suppressed by Fer-1. Significantly, an animal investigation showed that the administration of Fer-1 substantially enhanced the process of wound healing in diabetic rats [7]. Duscher *et al.* employed the Enhanced Concept Drug Delivery System (TDDS) to administer death inhibitors desferrioxamine (DFO) to the skin. Their findings revealed that diabetic mice exhibited notably elevated rates of wound healing, augmented neovascularization, and enhanced dermal thickness [8]. Exosomes containing cyclic atrophin 1 interacting protein 4 (ITCH) derived from bone marrow stromal cells (BMSC) are believed to suppress the expression of ferroptosis-associated proteins in human umbilical vein endothelial cells (HUVEC) that are induced by HG. This mechanism fosters the development of new blood vessels (angiogenesis) and expedites the healing of wounds in diabetic rats [151]. Moreover, platelet-rich plasma is thought to impede high glucose-induced ferroptosis, control the migration of fibroblasts and vascular endothelial cells, and augment the healing of diabetic wounds [51].

### 3.4 Antioxidant Pathways and Diabetic Wounds

The examination of the findings unveiled a positive correlation between serum ferritin levels and susceptibility to type 2 diabetes [152]. The investigation into DN indicated that individuals with DN displayed heightened levels of serum ferroptosis-related markers, specifically serum ferritin and lactate dehydrogenase (LDH), in comparison to healthy individuals. Li *et al.* observed a significant decrease in the expression levels of GPX4 and SLC7A11 in the renal tissues of diabetic mice compared to healthy mice. Conversely, the expression of TFR1 was shown to be increased in the renal tissues of diabetic mice [153]. Similar outcomes were observed in renal proximal tubular cells treated with HG. Notably, mesangial cells exhibited reduced expression of GPX4 and a significant decline in cell viability when exposed to HG conditions [96]. The p53 signaling pathway is activated in the aorta of db/db type 2 diabetic mice, resulting in malfunction of the systemic xc-. This dysfunction impacts the synthesis of glutathione and leads to de-endothelialization of the aorta [16]. Furthermore, *in vitro* research provided evidence that HG reduces the expression of GPX4 in endothelial cells by activating p53-xCT, which is the particular component of the systemic xc- -glutathione pathway. Tangeretin D, an anti-inflammatory compound, reversed the observed outcomes in HK-2 cells grown in high glucose by increasing the expression of GPX4 [6]. The iron DFO and Ferritin 1 (Fer-1) effectively reversed the process of endothelial cell death, indicating that HG triggers ferroptosis and subsequently results in endothelial dysfunction [132]. A laboratory-based study demonstrated that subjecting endothelial cells and fibroblasts to HG conditions resulted in a reduction in vascular VEGF levels in endothelial cells, impeded the migration of fibroblasts, and diminished the viability of both cell types. These effects were subsequently reversed by the administration of Fer-1. Fer-1 alleviated the decline in ferroptosis-associated markers GPX4 and SLC7A11, as well as the increase in TFR1 at both the transcriptional and translational levels in endothelial cells and fibroblasts under HG conditions [16]. There is an indication that the onset of renal ferroptosis in diabetic mice is associated with the reduction in the activity of the transcription factor NRF2 [153]. Oxidative stress is a crucial factor in the emergence of complications and impaired healing processes in individuals with diabetes. The transcription factor NRF2 directs the adaptive response to oxidative stress, prevents apoptosis, and promotes cell migration, proliferation, and differentiation [154]. The induction of NRF2 is initiated by heightened glucose levels and oxidative stress to manage and repair cellular damage. Long *et al.* showed that enhancing NRF2 activation reduces the level of oxidative stress induced by diabetes and regulates the expression of MMP-9, TGF-β, and genes related to migration and proliferation through direct or indirect mechanisms [155]. Significantly, HG-induced HK-2 exhibited elevated quantities of ROS, MDA, and ferrous Fe^2+^, indicating ferroptosis. These findings suggest that diabetes might heighten the susceptibility to ferroptosis and impede the process of wound healing [16]. At present, scholarly investigations have concentrated on the identification of therapeutics for diabetic wounds that target ferroptosis. Nevertheless, additional investigation is necessary to clarify the exact molecular mechanisms that underlie the connection between ferroptosis and the development of diabetic wounds. Furthermore, it is a prudent strategy to identify ferroptosis-associated genes that possess marker characteristics at various phases and within distinct cell populations throughout the progression of diabetic wounds. Lastly, systematic research is lacking to determine whether the function of ferroptosis differs throughout the body when diabetic wounds recover and how it evolves at various stages of the process. Further investigation into the correlation between ferroptosis and epigenetics, as well as intercellular communication, will contribute to a greater comprehension of the mechanism underlying the role of ferroptosis in diabetic wound healing [118]. This article presents a synopsis of the mechanisms involved in diabetic wounds and ferroptosis. It explores the role of ferroptosis-related granule proteins in diabetic wounds and summarizes recent research on the mechanisms associated with ferroptosis in diabetic wounds. Multiple laboratories have demonstrated that inhibiting excessive ROS formation and ferroptosis promotes diabetic wound healing. However, these investigations are still in their early exploratory phase, and there are numerous questions that warrant further scrutiny and inquiry [16].

## 4 WOUND HEALING ENHANCEMENT THROUGH FERROPTOSIS INHIBITION

In diabetic wound healing research, ferroptosis, a distinct process of cell death, offers novel therapeutic targets and avenues for investigation. Future treatment approaches may concentrate on the development and enhancement of ferroptosis inhibitors to mitigate oxidative stress and chronic inflammatory responses in diabetic wounds. Ferroptosis inhibitors, such as Ferrostatin-1 and Liproxstatin-1, have demonstrated encouraging outcomes in experimental settings, considerably enhancing the wound healing process by reducing lipid peroxidation and inflammatory mediator generation. Enhancements and alterations of these medications are anticipated to augment their safety and efficacy in clinical applications, particularly when administered topically to diabetic ulcerations, perhaps diminishing systemic adverse effects dramatically [156, 157].

Studies show that the persistent inflammation and oxidative stress in diabetic wounds can exacerbate delayed healing through iron-dependent lipid peroxidation. Cells in high-glucose environments exhibit elevated levels of ROS and lipid peroxidation products, triggering ferroptosis. Ferroptosis inhibitors, such as Ferrostatin-1, can improve diabetic wound healing by reducing the expression of inflammatory markers [7]. Moreover, chronic hyperglycemia leads to an accumulation of lipid peroxidation products and disrupts iron metabolism, further delaying wound healing and contributing to the underlying mechanism of diabetic ulcers [17]. Platelet-rich plasma (PRP) has been shown to significantly promote the healing of diabetic ulcers by inhibiting ferroptosis, reducing inflammatory factors, enhancing antioxidant defenses, and improving angiogenesis [51]. Additionally, autophagy-mediated reduction of Fe^2+^ production and regulation of iron metabolism help to improve cellular repair functions in diabetic wounds, thereby promoting the healing process [15]. Therefore, ferroptosis plays a crucial role in delayed diabetic wound healing, and strategies targeting its inhibition hold promise as new approaches and interventions for treating chronic diabetic wounds. These findings collectively suggest that the suppression of ferroptosis positively influences wound healing by reducing inflammatory responses, enhancing fibroblast migration and proliferation, and promoting angiogenesis, thereby improving overall tissue regeneration. The potential therapeutic application of ferroptosis inhibitors for treating diabetic wounds is promising, as they may offer an effective approach to managing chronic, non-healing wounds by addressing multiple physiological aspects of wound repair simultaneously.

Ferroptosis inhibition pathways play a crucial role in enhancing wound healing, especially in challenging conditions like diabetes. By inhibiting ferroptosis, these pathways prevent iron-dependent lipid peroxidation and reduce the accumulation of ROS, which are known to damage cells [17]. This preservation of cell integrity is particularly important for fibroblasts, keratinocytes, and endothelial cells, all of which are vital for wound repair processes. When ferroptosis is inhibited, the reduction in inflammation and oxidative stress creates a more favorable environment for tissue regeneration [153].

Moreover, novel methodologies integrating gene therapy and nanotechnology could augment the efficacy of ferroptosis modulation in wound healing. Gene editing approaches may modulate the expression of essential ferroptosis-related genes (*e.g.*, GPX4, SLC7A11), potentially intervening in cellular responses to oxidative stress at the molecular level, resulting in enduring and precise therapeutic effects. Simultaneously, nanocarriers may facilitate the targeted delivery of ferroptosis inhibitors to the wound site, thereby enhancing the local drug concentration and minimizing systemic toxicity. Future research should concentrate on the various pathologies of diabetic patients and integrate ferroptosis inhibition strategies with anti-inflammatory, antimicrobial, and angiogenesis-promoting therapies to develop a multifaceted treatment approach that offers a more comprehensive solution for challenging diabetic wounds.

In summary, the integration of ferroptosis inhibitors, gene therapy, and nanodelivery systems may transition the future therapeutic approach to diabetic wound management from conventional antibiotic and growth factor treatments to innovative strategies focused on cell death modulation, potentially enhancing wound healing and significantly improving the quality of life for diabetic patients.

## CONCLUSION

In summary, this article provides an overview of the molecular mechanisms involved in diabetic wound healing and explores the role of ferroptosis in this process. Ferroptosis can disrupt the functionality of skin constituent cells, including fibroblasts, KCs, ECs, and EPCs, as well as immune cells, such as macrophages and neutrophils, within diabetic ulcers. Consequently, diabetic lesions undergo compromised epithelialization, granulation, and inflammatory response, among other pathophysiological processes, leading to delayed wound closure. In the future, we can enhance the regulation of ferroptosis in clinical practice to facilitate diabetic wound healing by preventing iron insufficiency without causing excess, and by minimizing the generation of reactive oxygen species to prevent buildup. Ferroptosis has significant significance in preclinical investigations of various diseases, including cancer, neurological disorders, and conditions related to ischemia/reperfusion (I/R) injury. It presents a viable strategy for addressing drug-resistant cancer cells that have deficient apoptotic action. While blocking it can address infection-related disorders, aseptic inflammation linked to ferroptosis, or lipotoxicity. Modulating the ferroptosis pathway could mitigate the buildup of harmful byproducts, such as lipid peroxides and reactive aldehydes, perhaps decelerating neurodegeneration in multiple sclerosis and other disorders. I/R events induce oxidative stress and cellular apoptosis, rendering ferroptosis-targeted therapy promising for mitigating oxidative damage and maintaining tissue function in diseases like stroke, myocardial infarction, and renal and hepatic injuries. The simultaneous suppression of ferroptosis and necrotic apoptosis has demonstrated notable efficacy. In the renal tubule, the spread of ferroptosis has a distinctive pattern known as the “death wave,” which has since been observed in other systems. These results highlight the therapeutic potential of ferroptosis inhibitors in ischemia/reperfusion-related illnesses. The advancement of tailored medication delivery systems is crucial for enhancing treatment efficacy and reducing systemic side effects. Recent research has demonstrated potential in employing nanoparticles, such as liposomes, micelles, and polymer-based carriers, to tackle these issues. Nanoparticles have benefits, including improved drug stability, solubility, and targeted delivery. Further elucidation of the relationship between ferroptosis and diabetic wounds in the future holds the potential to contribute to the development of more effective treatments for diabetic wounds.

## Figures and Tables

**Fig. (1):**
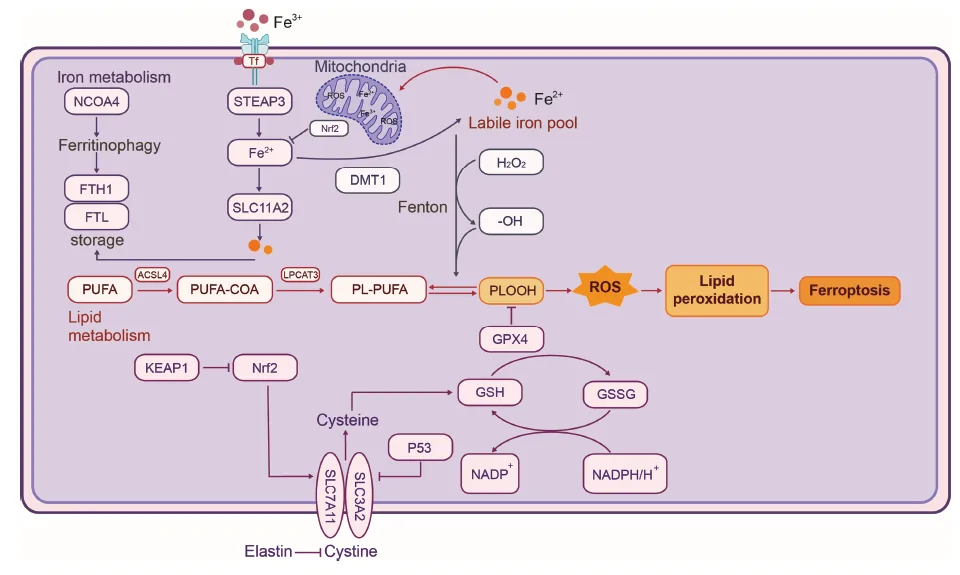
Illustrative depiction of the signaling pathways implicated in ferroptosis through lipid reactive oxygen species. Arrows represent the presence of promoter, whereas “T” symbols show the presence of inhibition. **Abbreviations:** TF: transferrin; NRF2: nuclear factor erythroid 2-related factor 2; MUFA: monounsaturated fatty acid; GSSG: glutathione disulfide; NADPH: nicotinamide adenine dinucleotide phosphate; NADP+: oxidized nicotinamide adenine dinucleotide phosphate; PUFA: polyunsaturated fatty acid; PUFA-CoA: polyunsaturated fatty acid acyl group; PL-PUFA: phospholipid-bound polyunsaturated fatty acid; PLOOH: phospholipid hydroperoxides; ACSL4: synthetase long-chain family 4; LPCAT3: lysophosphatidylcholine acyl group. phosphatidylcholine acyltransferase 3; FTL: Ferritin Light Chain; SLC11A2: Solute Carrier Cluster 11 member 2; KEAP1: Kelch-like ECH-associated protein. P53: Tumor suppressor protein; SLC7A11:Solute carrier family 7 member 11; SLC3A2: Solute carrier family 3 member 2.

**Fig. (2):**
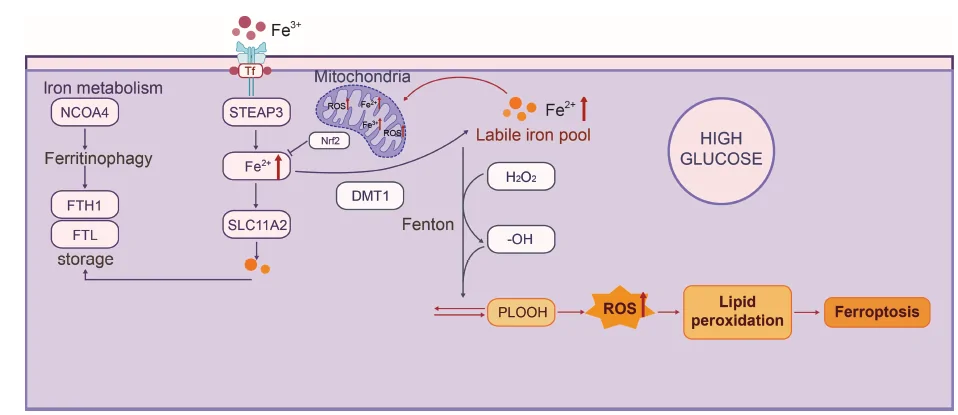
Effects of high glucose environment on proteins related to the ferroptosis mechanism. Arrows represent the presence of promoter, whereas“T” symbols show the presence of inhibition.

## LIST OF ABBREVIATIONS

AA: Arachidonic Acid
ADA: Adrenergic Acid
AIF: Apoptosis-inducing Factor
AIFM2: Factor Mitochondria-associated 2
AMID: Mitochondrion-associated Inducer of Death
Arg-1: Arginase-1
ATF3: Activating Transcription Factor 3
BMSC: Bone Marrow Stromal Cells
CD206: Cluster of Differentiation 206
CD86: Cluster of Differentiation 86
Coenzyme Q10: Coenzyme Quinone-10
DFO: Desferrioxamine
DN: Diabetic Nephropathy
DM: Diabetes Mellitus
ECs: Endothelial Cells
EPCs: Endothelial Progenitor Cells
Fer-1: Ferritin 1
Fe^2+^: Ferrous Iron
Fe^3+^: Ferric Iron
FSP1: Ferroptosis Suppressor Protein 1
FTL: Ferritin Light Chain
FTH1: Ferritin Heavy Chain 1
GPX4: Glutathione Peroxidase 4
GPxs: Glutathione Peroxidase
GSH: Glutathione
GSSG: Glutathione Disulfide
HG: High Glucose
HDF: Human Dermal Fibroblasts
HMGB1: High Mobility Group Box Chromosomal Protein 1
HUVEC: Human Umbilical Vein Endothelial Cells
HK-2 cells: Renal Tubular Cells
IL-1β: Interleukin-1β
IL-6: Interleukin-6
ITCH: Atrophin 1 Interacting Protein 4
KEAP1: Kelch-like ECH-associated Protein
KCs: Keratinocytes
LPCAT3: Lysophosphatidylcholine Acyl group. Phosphatidylcholine Acyltransferase 3
LDH: Lactate Dehydrogenase
LIPs: Labile Iron Pool
LOOH: Lipid Hydroperoxides
LOX: Lipoxygenase
MDA: Malondialdehyde
MUFA: Monounsaturated Fatty Acid
NADP+: Oxidized Nicotinamide Adenine Dinucleotide Phosphate
NADPH: Nicotinamide Adenine Dinucleotide Phosphate
NCOA4: Nuclear Receptor Coactivation Factor 4
NRF2: Nuclear Factor Erythroid 2-related Factor 2
PCNA: Proliferating Cell Nuclear Antigen
PL-PUFA: Phospholipid-bound Polyunsaturated Fatty Acid
PLOOH: Phospholipid Hydroperoxides
PRG3: Recombinant p53 Responsive Gene 3
PUFA: Polyunsaturated Fatty Acid
PUFA-CoA: Polyunsaturated Fatty Acid Acyl Group
ROS: Reactive Oxygen Species
SLC11A2: Solute Carrier Cluster 11 Member 2
SLC3A2: Solute Carrier Family 3 Member 2
SLC7A11: Solute Carrier Family 7 Member 11
STEAP3: Six Transmembrane Epithelial Antigen 3
T2DM: Type 2 Diabetes Mellitus
TCA: Tricarboxylic Acid Cycle
TDDS: Concept Drug Delivery System
TfR1: Transferrin Receptor 1
TF: Transferrin
TGF-β: Transforming Growth Factor-β
TNF-α: Tumor Necrosis Factor-α
TP53: Tumor Suppressor Protein
VEGF: Vascular Endothelial Growth Factor
